# Is Universal HBV Vaccination of Healthcare Workers a Relevant Strategy in Developing Endemic Countries? The Case of a University Hospital in Niger

**DOI:** 10.1371/journal.pone.0044442

**Published:** 2012-09-07

**Authors:** Gérard Pellissier, Yazdan Yazdanpanah, Eric Adehossi, William Tosini, Boubacar Madougou, Kaza Ibrahima, Isabelle Lolom, Sylvie Legac, Elisabeth Rouveix, Karen Champenois, Christian Rabaud, Elisabeth Bouvet

**Affiliations:** 1 Groupe d’Etude sur le Risque d’Exposition des Soignants aux agents infectieux (GERES), Faculté de Médecine Paris VII - Bichat, Paris, France; 2 Service de Maladies Infectieuses et Tropicales, Hôpital Bichat-Claude Bernard, Assistance Publique-Hôpitaux de Paris, Paris, France; 3 ATIP-Avenir Inserm, Modélisation, Aide à la Décision, et Coût-Efficacité en Maladies Infectieuses, Lille - Paris, France; 4 Département de Médecine, Hôpital National de Niamey, Niamey, Niger; 5 GIP-ESTHER, Niamey, Niger; Paris, France; 6 Unité d’Hygiène et de Lutte contre l’Infection Nosocomiale, Hôpital Bichat-Claude Bernard, Assistance Publique-Hôpitaux de Paris, Paris, France; 7 Entraide Santé 92, Vaucresson, France; 8 Service de Médecine, Hôpital Ambroise Paré, Assistance Publique-Hôpitaux de Paris, Boulogne-Billancourt, France; 9 Service de Maladies Infectieuses, Centre Hospitalier Universitaire, Nancy, France; INSERM U1094, University of Limoges School of Medicine, France

## Abstract

**Background:**

Exposure to hepatitis B virus (HBV) remains a serious risk to healthcare workers (HCWs) in endemic developing countries owing to the strong prevalence of HBV in the general and hospital populations, and to the high rate of occupational blood exposure. Routine HBV vaccination programs targeted to high-risk groups and especially to HCWs are generally considered as a key element of prevention strategies. However, the high rate of natural immunization among adults in such countries where most infections occur perinatally or during early childhood must be taken into account.

**Methodology/Principal Findings:**

We conducted a cross sectional study in 207 personnel of 4 occupational groups (medical, paramedical, cleaning staff, and administrative) in Niamey’s National Hospital, Niger, in order to assess the prevalence of HBV markers, to evaluate susceptibility to HBV infection, and to identify personnel who might benefit from vaccination. The proportion of those who declared a history of occupational blood exposure ranged from 18.9% in the administrative staff to 46.9% in paramedical staff. Only 7.2% had a history of vaccination against HBV with at least 3 injections. Ninety two percent were anti-HBc positive. When we focused on170 HCWs, only 12 (7.1%) showed no biological HBV contact. Twenty six were HBsAg positive (15,3%; 95% confidence interval: 9.9%–20.7%) of whom 8 (32%) had a viral load >2000 IU/ml.

**Conclusions/Significance:**

The very small proportion of HCWs susceptible to HBV infection in our study and other studies suggests that in a global approach to prevent occupational infection by bloodborne pathogens, a universal hepatitis B vaccination of HCWs is not priority in these settings. The greatest impact on the risk will most likely be achieved by focusing efforts on primary prevention strategies to reduce occupational blood exposure. HBV screening in HCWs and treatment of those with chronic HBV infection should be however considered.

## Introduction

Hepatitis B virus (HBV) infection is one of the most frequent human diseases with more than 350 millions people chronically infected with the HBV worldwide [1]. Of these, at least one million die each year from chronic liver disease. The prevalence of infection varies by geographic region and approximately 45% of the world’s population live in areas of high HBV endemicity [2] with population prevalence ≥8%: Sub-Saharan Africa, Pacific, and Asia. The implementation of mass immunization programs has been recommended by the WHO and has dramatically decreased the incidence of HBV infection [3]–[5] and also poliomyelitis, diphteria, tetanus, measles, pertussis, tuberculosis, among infants, children and adolescents in many countries. In Niger, Hepatitis B vaccination has been included in the Expanded Program on Immunisation in 2008, targeting children. Following this program national vaccination coverage rate was estimated to be 70% in one-year old children (WHO/UNICEF estimates, 2009). However, this vaccination program does not yet have any impact on the current Nigerian general population immunisation profile.

HBV is a bloodborne virus and is transmitted by both percutaneous and mucosal exposures [6], [7]. HBV rate of transmission after percutaneous injury was estimated to be 100 times higher than for HIV and 10 times more transmissible than HCV: 3–30% depending on source patient’s viral load [8]. Immunization of healthcare workers (HCWs) against HBV assumes great importance and is universally recommended [9]–[11]. Various studies [3]–[5] have demonstrated the efficacy of vaccination to prevent HBV infection. Nevertheless, in endemic developing countries, particularly in Sub-Saharan Africa, few HCWs are vaccinated adequately and even fewer are screened [12]–[14].

Exposure to HBV remains a serious risk to HCWs owing to the strong prevalence of HBV in the general and hospital populations, and to the high rate of needlestick injury when compared to industrialized countries [14]–[18]. The World Health Organization (WHO) has estimated in a model-based analysis that 6200 HBV infections occur each year among sub-Saharan African HCWs [16], based on an estimated vaccine coverage rate of 18%. Routine HBV vaccination programs targeted to high-risk groups and especially to HCWs are thus generally considered as a key element of prevention strategies in such endemic developing countries [12], [16], [19]–[21]. However, these considerations did not take into account the high rate of natural immunisation among adults in endemic countries, and especially in sub-Saharan Africa, where most infections occur perinatally or during early childhood [22], [23].

The French ESTHER initiative aimed at creating a hospital network for therapeutic solidarity between Northern and Southern hospitals, in order to facilitate access to care for people living with HIV/AIDS in developing countries. Within this initiative and hospital network the GERES Research Group conducted in Niamey’s National Hospital (NNH), Niger, a project to reduce needlestick injuries and infectious risks among HCWs. A study was undertaken in 2009, as part of this project, in order to assess the prevalence of HBV markers among HCWs in NNH, to evaluate HCWs susceptibility to HBV infection, and to identify categories and numbers of HCWs who might benefit from vaccination.

## Methods

Ethics statements: the protocol of this study has been submitted and approved by the Niger ethics committee (Comité Consultatif National d’Ethique). All workers involved in the study have given prior written informed consent.

We used a cross sectional study design. Because Romieu et al. [24] found that 17.8% of HCWs of the Dakar hospital, Senegal, were HBsAg carriers, we anticipated a prevalence of HBsAg marker close to 15% in NNH staff. To have a precision of 5% around the estimated prevalence, the calculated sample size was 196 subjects. NNH had 829 employees, so a quarter of them were selected randomly according to the occupational category.

Workers of four occupational groups (medical, paramedical, cleaning staff – involved in the disposal of waste, and administrative) were selected by random sampling from a categorized list obtained from the hospital administration, proportionate to the population of each occupational group in NNH. The first three groups were representative of HCWs (and other at-risk personnel). Eligible and sampled individuals who turned down participation were replaced with individuals randomly picked from the same occupational category. All but one contacted subjects participated in the study.

Socio-demographic characteristics (gender, age, birthplace), the presence of HBV infection risk factors (presence of family HBV carriers, history of jaundice, occupational blood exposure) and history of vaccination were assessed through a standardized questionnaire during the initial medical consultation. Blood samples obtained from each participant were tested for HBV markers: Anti-HBc, if reactive, HBsAg, if reactive, HBeAg and HBV DNA; if Anti-HBc non reactive, Anti-HBs. Non-immune HCWs were offered hepatitis B vaccination.

HBsAg and Anti-HBc were detected, and anti Anti-HBs quantification performed, using the AxSym immunoenzymatic assay (Abbott Laboratories). An Anti-HBs titre of 10 IU/L or greater was considered to confer protectivity. These assays were conducted in the biology laboratory of NNH. Quantification of viral DNA was performed using a real-time PCR assay (CAP/CTM TaqMan, Roche Laboratories) and conducted in the National Reference Laboratory for tuberculosis and Aids, Lamordé National Hospital, Niamey.

The statistical analysis was descriptive. Estimated prevalence of HBV markers are presented with a 95% confidence interval (95%CI). The Pearson Chi-square test, Fisher exact test and Cochrane-Armitage trend test were used for analyses. Calculations were performed using Epi-info (Version 6.04d, Centers for Disease Control and Prevention, Atlanta, GA, USA) and SAS (Version 9.2, SAS Institute, Cary, NC, USA) softwares.

## Results

In total, 207 personnel were included and their characteristics are presented in Table 1. These personnel mean age was 40±10 years (range 20–62 years) and 51% were men. Of these 11.6% were doctors (n = 24), 54.6% paramedicals (n = 113), 15.9% cleaning staff (n = 33), and 17.9% administrative personnel (n = 37). 7.2% (15/207) had a history of vaccination against HBV with at least 3 injections. Among these only 2 had a serological profile attesting vaccination (presence of HBs antibodies only); the other 13 had a serological profile showing natural immunity to the disease (presence of HBc antibodies).

**Table 1:** Characteristics of personnel (n = 207) at Niamey’s National Hospital, Niger, 2009.

Characteristics,		All personnel	Anti-HBc (+)
n (%)		(N = 207)	(N = 191)
Age (years)			
	20–29	31 (15.0)	27 (14.1)
	30–39	73 (35.3)	66 (34.6)
	40–49	55 (26.6)	51 (26.7)
	50 and above	43 (20.8)	42 (22.0)
	Unknown	5 (2.4)	5 (2.6)
Sex			
	Female	101 (48.8)	92 (48.2)
	Male	106 (51.2)	99 (51.8)
Country of birth			
	Niger	192 (92.8)	178 (93.2)
	Other Countries	15 (7.2)	13 (6.8)
Occupational category			
	Medical staff	24 (11.6)	19 (9.9)
	Paramedical staff	113 (54.6)	105 (55.0)
	Cleaning straff	33 (15.9)	32 (16.8)
	Administrative staff	37 (17.9)	35 (18.3)
History HBV vaccination			
	No	160 (77.3)	148 (77.5)
	Yes, ≥3 doses	15 (7.2)	13 (6.8)
	Yes, <3 doses	19 (9.2)	18 (9.4)
	Yes, unknown number of doses	8 (3.9)	8 (4.2)
	Unknown	5 (2.4)	4 (2.1)
History occupational blood exposure			
	Yes	83 (40.1)	78 (40.8)
	No	116 (56.0)	106 (55.5)
	Unknown	8 (3.9)	7 (3.7)

The proportion of those who declared a history of occupational blood exposure ranged from 18.9% in the administrative staff to 46.9% in paramedical staff. Histories of occupational blood exposure among different occupational categories are presented in Table 2. Administrative staff, some of whom were previously HCWs (presumably having been exposed for a shorter period of time), were less likely to report a history of occupational blood exposure than medical or paramedical staffs. Moreover, health workers (medical, paramedical, and cleaning staffs) age 40 and above more frequently reported an occupational blood exposure history than younger ones, age 20–39 (54.1% vs 36.3%; p = 0.016).

**Table 2:** History of occupational blood exposure (OBE) among Health workers of different occupational categories compared to administrative personnel at Niamey’s National Hospital, Niger, 2009.

Occupational category	History of OBE, n (%)			
	Yes	No or Unknown	RR (95% CI)	p Value
Paramedical staff	53 (63.9)	60 (48.4)	2.48 (1.24–4.97)	0.002
* Nurses*	*31 (37.3)*	*29 (23.4)*	*2.73 (1.34–5.56)*	*0.001*
Medical staff	11 (13.3)	13 (10.5)	2.42 (1.09–5.37)	0.02
Cleaning staff	12 (14.5)	21 (16.9)	1.92 (0.86–4.30)	0.10
Administrative staff	7 (8.4)	30 (24.2)	1	
Total	83 (100)	124 (100)		

RR, relative risk; 95% CI, 95% confidence interval.

Among those enrolled in this study 92.3% were anti-HBc positive (191/207). The prevalence of Anti-HBc was not found to be associated to age, sex, occupational category, history of HBV vaccination, or history of occupational blood exposure. When we focused on 170 HCWs (Table 3), excluding administrative personnel, 26 were HBsAg positive (15.3%; 95% CI: 9.9–20.7) of which all but one (96%) were HBeAg negative. Of these, 8 (32%) had a viral load >2000 UI/ml (range 2,520–2,080,000). One hundred twenty nine (75.9%; 95% CI: 69.5–82.3) were anti-HBc positive and HBsAg negative and therefore had an evidence of natural immunization. Three HCWs were immune due to a vaccination (1.8%; 95%CI: 0–3.8). Among them, 2 declared a history of HBV vaccination with at least 3 doses, and one said he could not remember an history of HBV vaccination. Only 12 personnel were anti-HBc negative and anti-HBs negative (7.1%; 95% CI: 3.2–11.0) and therefore showed no previous HBV contact. In the 37 administrative personnel, 1 (2.7%; 95% CI:0–7.9-) showed no previous HBV contact. The prevalence of immunity due to natural infection (resistance to infection because of previous exposure to HBV naturally: Anti-HBc positive and HBsAg negative) by age group is presented in Table 4. The proportion of HCWs who were 20 to 24 years old and who were already immune to hepatitis B was 71% and this proportion was stable until 40 years of age. There was a trend toward an increase in the immunity over but this trend was not statistically significant (p = 0.07).

**Table 3:** HBV serology in personnel (n = 207) at Niamey’s National Hospital, Niger, 2009.

Serological test, n (%;95% CI)				All personnel	Healthcare staff	Administrative	P
Anti-HBc	HBsAg	Anti-HBs	Interpretation	(N = 207)	(N = 170)	staff (N = 37)	Value
Pos	Neg	–	Immunity due to natural infection (previously infected)	161 (77.8;72.1–83.5)	129 (75.9;69.5–82.3)	32 (86.5;75.5–97.5)	0.16
Pos	Pos	–	Currently infected	30 (14.5;9.7–19.3)	26 (15.3;9.9–20.7)	4 (10.8;0.8–20.8)	0.34
Neg	–	Neg	Susceptible to HBV infection(not infected)	13 (6.3;3.0–9.6)	12 (7.1;3.2–11.0)	1 (2.7;0–7.9)	0.29
Neg	–	Pos	Immunity due to HBVvaccination (not infected)	3 (1.4;0–3.0)	3 (1.8;0–3.8)	0	0.55

Pos, seropositive; Neg, seronegative; –, not performed; 95%CI, 95% confidence interval.

**Table 4:** Prevalence of immunity due to natural infection (previously infected; Anti-HBc positive and HBsAg negative) by age group in personnel at Niamey’s National Hospital, Niger, 2009.

Age (year)	Immunity due to natural infection, n		% immune	P* Value = 0.07
	Immune	Non Immune		
20–24	5	2	0.71	
25–29	18	6	0.75	
30–34	26	11	0.70	
35–39	25	11	0.69	
40–44	29	4	0.88	
45–49	18	4	0.82	
50–54	20	5	0.80	
55 & above	16	2	0.89	
*Unknown*	*4*	*1*		
Total	161	46	0.78	

* Chi2 trend test.

## Discussion

This study shows that the anti-HBc antibodies prevalence is extremely high in this National Hospital in a large sub-Saharan African city versus 4.7% in the USA, for example [25]. Other surveys among sub-saharan African HCWs have shown a comparable HBV markers prevalence. For example Hepatitis B exposure assessed in HCW in Uganda showed that only 36% were still susceptible and could benefit from vaccination [26]. The anti-HBc antibodies prevalence was 79% in 775 hospital workers in hospitals in Dakar, Senegal and 62% in surgeons in a major city in Nigeria [24], [27]. In our study, data on prevalence of immunity due to natural infection showed that a high proportion of young HCWs who entered work at health care facilities were already immune against hepatitis B supporting the fact that exposure occurred before entering work. These data are consistent with those from other studies conducted in various groups in Niger [28]–[31]. A serological study of hepatitis B conducted in 1983 in people leaving in villages in Niger showed 19% were positive for HBsAg. When Anti-HBc was included, 82% were found to be positive for at least one virus marker. This latter proportion increased with age, from 53% in 1–4 year old subjects to 98% in subjects over thirty years of age [32]. Moreover, in our study the proportion of susceptibles that could benefit from HBV vaccination was low even in the administrative personnel. Most HBV infections in this setting likely occured perinatally or secondarily during early childhood. The very low number of HCWs susceptible to HBV infection in our study and other studies suggests that in a global approach to prevent occupational infection by bloodborne pathogens, a universal hepatitis B vaccination of HCWs may not be the priority in these settings. Compulsory HBV vaccination in childhood remains the optimal immunization policy to interrupt transmission in HBV endemic countries [33]–[35]. This maybe interesting in those starting medical or paramedical schools or first starting to work in a health care setting but this needs to be further evaluated.

Of note this study demonstrated the absence of suspicions expressed by HCWs about giving blood for analysis, and the feasibility and acceptability of prevaccination screening strategy. Following the first information campaign, 256 personnel, in addition to those enrolled in this study went to medical consultation of which 32 were receptive to vaccination and have been vaccinated. Nevertheless, testing of HCWs may have other ethical implications if positive HCWs, whose number is not negligible, are to have restrictions placed on their practice, and this measure is likely to weaken health systems already fragile in resources constrained endemic countries.

In another hand, HCWs preventive interventions to prevent needlestick injuries should be however conducted due to the high rate of occupational blood exposure faced by HCWs in this setting and more generally in endemic developing countries. These interventions may be more effective and cost-effective than universal vaccination campaigns because preventing also other bloodborne pathogens as HCV and HIV although this needs to be demonstrated in a formal analysis. The greatest impact on the risk of bloodborne pathogens transmission to HCWs will most likely be achieved by focusing efforts on primary prevention strategies to reduce the infectious risk for transmission from occupational blood exposure. Resources not spent for HCWs HBV universal vaccination could be dedicated to campaigns to promote adherence to standard precautions, to implement standard barrier precautions such as gloves, gowns and eyewear, to use appropriate measures to prevent percutaneous injuries (eliminating unnecessary injections, avoidance of recapping needles, appropriate disposal of sharps in safety containers) and use of safer needle devices such as safety engineered devices for example. These measures could reduce the transmission of all bloodborne pathogens, including HBV for the few susceptible HCWs.

The high prevalence of HCWs with positive HBs Ag (15.3%; 95% CI: 9.9–20.7), and among them the high proportion of those with a viral load >2000 IU/ml raise the question of HBV screening in HCWs to identify personnel with chronic hepatitis B. The potential for transmission of bloodborne pathogenes, and particularly HBV which is more highly transmissible than HIV or hepatitis C virus, from infected HCW to patients is well documented and is an important issue facing healthcare policymakers internationally [36], [6]. HBV screening in HCWs could offer the possibility to detect chronic carriage of HBV, with the feasibility of providing treatments in resource-limited countries, such as Tenofovir, entecavir or telbivudine active against HBV [37]. Antiviral treatment is the only way to reduce morbidity and mortality from chronic HBV infection. The benefits of such efficacious treatments have been clearly established and a high proportion of persons do respond to currently available treatment [38]. Identifying and treating infected and viremic HCWs will reduce HCW to patient transmission of bloodborne pathogens.

### Conclusions

The very small proportion of HCWs susceptible to HBV infection in our study and other studies suggests that in a global approach to prevent occupational infection by bloodborne pathogens, a universal hepatitis B vaccination of HCWs is not priority in resources constrained settings in HBV endemic countries. The greatest impact on the risk of bloodborne pathogens transmission will most likely be achieved by focusing efforts on primary prevention strategies to reduce the infectious risk for transmission from accidental blood exposure. These measures could reduce the transmission of all bloodborne pathogens, including HBV for the few susceptible HCWs but also other pathogens such as HCV and HIV.
